# The La-Related Proteins, a Family with Connections to Cancer

**DOI:** 10.3390/biom5042701

**Published:** 2015-10-16

**Authors:** Chara Stavraka, Sarah Blagden

**Affiliations:** 1 Ovarian Cancer Research Centre, Institute for Reproductive and Developmental Biology, Imperial College, Du Cane Road, London W12 0HS, UK; E-Mail: chara.stavraka09@imperial.ac.uk; 2 Department of Oncology, University of Oxford, Churchill Hospital, Old Road, Oxford OX3 7LE, UK

**Keywords:** cancer, LARP, LARP1, transcription, mRNA, translation, proliferation, RNA-binding, RBP, SS-B

## Abstract

The evolutionarily-conserved La-related protein (LARP) family currently comprises Genuine La, LARP1, LARP1b, LARP4, LARP4b, LARP6 and LARP7. Emerging evidence suggests each LARP has a distinct role in transcription and/or mRNA translation that is attributable to subtle sequence variations within their La modules and specific C-terminal domains. As emerging research uncovers the function of each LARP, it is evident that La, LARP1, LARP6, LARP7 and possibly LARP4a and 4b are dysregulated in cancer. Of these, LARP1 is the first to be demonstrated to drive oncogenesis. Here, we review the role of each LARP and the evidence linking it to malignancy. We discuss a future strategy of targeting members of this protein family as cancer therapy.

## 1. Introduction

Cancer is a disease on the rise, from a current worldwide incidence of 14 million cases per year to a predicted 25 million cases per year by 2030 [1]. In developed countries, this reflects an increase in average longevity. In developing countries, rising cancer incidence is due to greater Westernization: more tobacco use, increased consumption of alcohol and processed food along with less physical exercise. However, in these countries, there remains a persistence of infection-associated cancers (such as cervical cancer) due to lack of screening and disease-prevention programmes [2]. Worldwide, apart from the recent discovery of immunotherapy strategies effective in melanoma and lung cancer [3], chemotherapy has remained the mainstay of systemic therapy since its introduction in the 1940s. This is despite the information revolution that followed publication of the human genome sequence in 2001, against which the genomes of common cancers were compared leading to the development of specific, molecularly-targeted anti-cancer agents [4,5]. Although these drugs have dramatically improved survival from malignancies with driver mutations like HER2-positive breast cancer [6,7], melanoma [8] and renal cell cancer [9], their impact on others such as cancers of the stomach, brain, oesophagus and lung has been negligible [10]. In these and many other cancers, there is a requirement for new treatment strategies.

In the shadow of the genomic revolution, another has occurred, that of RNA biology. In the last decade, microRNAs (miRs), non-coding RNAs (ncRNAs) and RNA-binding proteins (RBPs) have been recognised as having a dominant influence over gene expression [11,12]. Improvements in RNA capture and sequencing techniques have identified RBPs as important post-transcriptional regulators, as they control protein expression from multiple mRNAs simultaneously [11,13]. The first RBP to be linked to cancer was eukaryote initiation factor 4E (eIF4E), when investigators demonstrated that its ectopic expression could drive malignant transformation in fibroblasts and mammalian epithelial cells [14]. Elevated levels of phosphorylated eIF4E within a tumour are associated with resistance to cell stress and DNA-damaging agents and, as with expression of its binding partner 4E-BP1, with adverse survival outcome in multiple human cancers [15,16,17]. The discovery that the anti-viral drug ribavirin has eIF4E inhibitory activity has resulted in its re-appropriation as a cancer therapy [18]. Here, we will discuss an evolutionarily conserved family of RBPs, the La related proteins (LARPs) that are associated with cancer.

## 2. The LARP Family—An Overview

**Figure 1:**
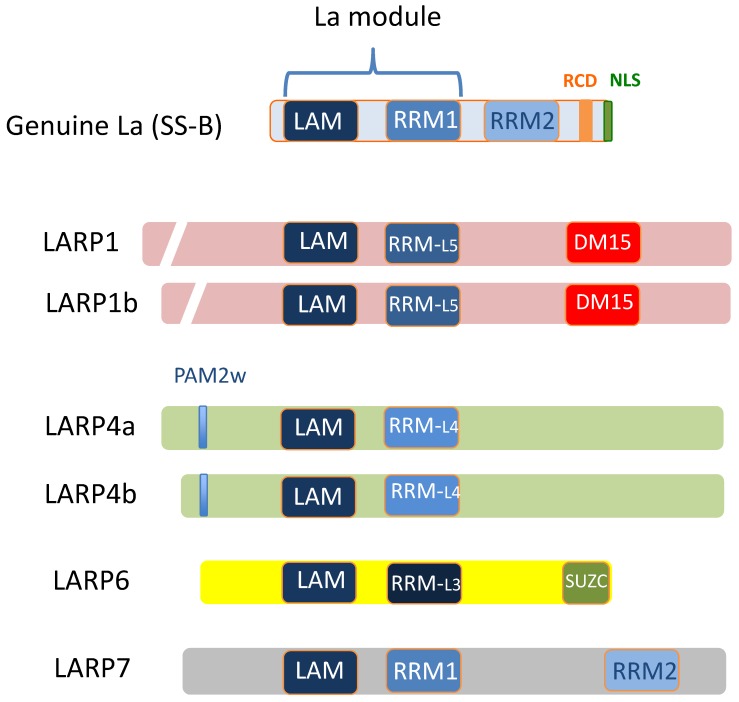
The principal domains common to members of the LARP protein family. Abbreviations: DM15: DM15-repeat containing region (“DM15 region”) also known as “LARP1 motif”; LAM: La Motif; NLS: Nuclear localisation signal; PAM2w: Atypical PAM2 domain; RCD: RNA chaperone domain; RRM: RNA Recognition Motif; RRM-L: RNA recognition-like motif; SUZ-C: SUZ-C domain. (Modified from Bayfield & Maraia, 2010 [20])*.*

Members of the LARP family are so named because they carry a conserved 90-amino acid signature La motif (LAM) similar to that of Genuine La protein. LARPs are an ancient family of proteins, conserved throughout eukaryote evolution. Humans carry five LARP subfamilies: Genuine La (previously known as SS-B and recently termed LARP3 by HUGO gene classification), LARP1 (variants 1a and 1b), LARP4 (variants LARP4a and 4b), LARP6 and LARP7. The evolution and structural characteristics of the LARP family have been described in reviews by both Bousquet-Antonelli & Deragon [19] and Maraia *et al.* [20] and summarised in Figure 1. LARP family members lack enzymatic domains but carry a LAM alongside an adjacent RNA recognition motif (RRM) and these two motifs are collectively termed the “La Module”. The placement of the La module differs between LARP proteins, in Genuine La it is located near the N-terminus, but for the other LARPs, it is positioned more centrally. The RRM within the La module is canonical in Genuine La and LARP7 but for the other LARPs it is a non-typical “RRM-like” region. Genuine La and LARP7 also carry a second RRM, the RRM2, which is absent from other LARP members. In the LARP1 subfamily, there is a C-terminally placed motif comprised of triplicate amino acid repeats. This was originally named the “DM15 region” [21] but is now termed the “LARP1 motif” in recognition that it is unique to LARP1 [19]. In contrast, LARP6 carries a 36 amino acid SUZ-C motif in its extreme C-terminus. This domain has been observed in the C-termini of other RBPs and is believed to be required for their subcellular localisation [22]. To our knowledge, LARP4a and b do not carry any additional C-terminal motifs but have an atypical N-terminal PAM2 domain (the PAM2w) to bind polyadenylate binding protein (PABP) [23].

The first LARP family member to be described was Genuine La and other members of the LARP family have subsequently been characterised [19]. Each has been shown to have roles in transcription and/or translation. Table 1 provides an “at-a-glance” summary of the LARPs.

**Table 1:** A summary of our current knowledge of the LARP family of proteins. Putative isoform information was obtained from GeneCards^®^ database [24].

Properties	Genuine La	LARP1	LARP1b	LARP4a	LARP4b	LARP6	LARP7
**Pseudonyms**	SS-B, LARP3	LARP1a	LARP2	LARP4	LARP5, KIAA0217	Acheron, ACHN	PIP7S, HDCMA18
**First time described in any organism**	1976 (Alspaugh *et al.*) [25]	2000 (Chauvet *et al.*) [26]	2002 (Wolin & Cedervall) [27]	2011 (Yang *et al*.) [23]	2002 (Angenstein *et al.*) [28]	2007 (Valavanis *et al.*) [29]	2008 (Krueger *et al.*) [30]
**Number of isoforms** (from NCBI)	2	3	9	7	2	2	3
**Size of main isoform** (aa = amino acids)	408 aa	1096 aa	914 aa	724 aa	738 aa	491 aa	582 aa
**Molecular mass of main isoform**	46 kDa	123 kDa	105 kDa	80 kDa	80 kDa	54 kDa	66 kDa
**Role in transcription?**	Yes Protect PolIII transcripts [27], maturation of pre-tRNA and noncoding RNAs [31,32]	-	-	-	-	Yes Interacts with Id transcription factors, vimentin, non-muscle myosin MYH10, DHX9 [33,34]	Yes Binds 7SK snRNP and negatively regulates RNA pol II transcription [35]
**Role in translation?**	Yes IRES mediated [36], 5'TOPs (repression) [20]	Yes Regulates the stability and/or translation of TOP mRNAs and others [37,38]	-	Yes Promotes mRNA stability [23]	Yes Stimulates translation and circularises mRNAs [39]	Yes Promotes translation of collagen [40]	-
**Known mRNA targets**	5'TOPs and IRES-mRNAs [20,41,42]	>3000 mRNAs including 5'TOPs and mTOR [38,43]	-	Single stranded poly(A) stretches [23]	-	Type I collagen [33,40]	-
**Confirmed protein binding partners**	-	Raptor, eIF4E, eIF4A, PABP, 5'TOP mRNAs [37,38,44,45,46]	-	RACK1, 40S components [23]	RACK1, 40S components [23]	Vimentin intermediate filaments, RNA helicase, STRAP, non-muscle myosin CASK, ID factors [47]	MePCE [30,48]
**Substrate recognition motifs**	5' Stem loop in IRES mRNAs, 3' end of PolIII transcripts, stem loop of miRNAs	5' end of TOPs, 3' end of BCL2, BIK—recognition sequence or structure unknown	-	-	-	5' stem loop in alpha collagen	3' end of 7SK RNA
**PABP interaction demonstrated?**	no	yes	-	yes	yes	Yes	no
**3D structure known?**	La module bound to 3'UUU-OH [49,50,51]	DM15/LARP1 region [52]	-	PAM2 [23]	-	La module [20,29]	La module [53]
Cancers associated (* *in vitro*)	Head and neck *, cervix *, liver *, myeloproliferative * [36,41,54,55]	Cervix *, liver, breast *, non small cell lung cancer *, prostate * [43,44,56,57]	-	Prostate cancer * [58]	Acute myeloid leukaemia * [59]	Breast cancer * [60]	Cervix *, gastric *, breast * [61,62,63]
**Tumour suppressing or oncogenic?**	Proto-oncogenic	Proto-oncogenic	-	Tumour suppressing (preliminary)	Proto-oncogenic	Proto-oncogenic	Tumour suppressing
**Drug target**	-	-	-	-	-	Yes—for fibrotic disease [64]	-

## 3. Genuine La

Human La protein was first identified as SS-B, an autoantigen expressed in immune disorders [25,65]. Levels of circulating anti-La antibody are used to diagnose those with auto-immune Sjogren’s syndrome, systemic lupus erythematosus (SLE) and neonatal lupus syndrome [66,67]. At 46kDa, genuine La is the smallest member of the LARP superfamily but is its most abundant [27]. Its LAM adopts a winged-helix type configuration [49,68] commonly seen in DNA transcription factors. The LAM co-operates with the adjacent RRM that, along with the connecting linker region, creates an RNA-binding pocket. This “La module” acts as a single functional unit and folds around the 3' UUU_OH_ termination motifs of nascent, misfolded RNA polymerase III transcripts (including pre-transfer RNAs (tRNAs)) to ensure correct folding and to protect them from exonuclease digestion [27]. The recognition of the UUU-3-OH terminus within these target transcripts is sequence-specific, requiring two or three U residues and a free-OH group at its 3' end [69]. With strand-annealing and dissociation capabilities, La is also believed to act as a pre-tRNA chaperone even to non UUU-3'OH bearing targets [31,32,70,71]. This indicates that, beyond its remedial function of correcting misfolding, La has a fundamental role in tRNA assembly. La has recently been shown to contribute to miRNA processing by associating with nascent pre-microRNAs (miRs), protecting them from nuclease digestion. Interestingly, the majority of these pre-miRNAs do not carry poly-UUU tails, but La recognises their characteristic stem loop conformation. It has also been shown that, whilst the La module is a prerequisite for 3'UUU_OH_ and pre-tRNA binding, the entire LAM-RRM1-RRM2 stretch is required for its non-3'UUU_OH_ interactions [72].

As well as transcription, La is involved in mRNA translation where it associates with the opposite, 5' end, of transcripts. La is deployed during viral mRNA translation [73], an area of research beyond the scope of this review. In conditions of cellular stress in which the predominant cap-mediated means of mRNA translation is abrogated, cytoplasmic La acts as an internal ribosome entry site (IRES) trans-acting factor (ITAF) [36]. Although the exact site of interaction remains elusive, La recognises stem-loop structures in or around the start codon of target mRNA transcripts. Not only does La attract ribosomes to initiate IRES-mediated translation but it has also been shown to unwind and reconfigure the IRES binding site, the latter function attributed to the RNA chaperone domain (RCD) within its C-terminus [74]. Examples of IRES mRNAs regulated by La include Cyclin D1 [41], XIAP [75], the chaperone immunoglobulin heavy chain binding protein BiP and the cell cycle activator Murine Double Minute 2 (MDM2) [42,76]. Whilst La is recognised to positively regulate IRES translation, its role in the translation of 5' terminal oligopyrimidine (TOP) containing mRNAs has been more controversial. TOP mRNAs are so named because they carry a 4–14 stretch of pyrimidine residues followed by a GC-rich region immediately adjacent to their 5' m^7^Gppp cap. They encode proteins required for the synthesis of ribosomes and other components of the translational apparatus (ribosome biogenesis) to ensure the cell can meet the demands of cell growth or proliferation [77]. Work in *Xenopus* initially characterised La as an activator of 5'TOP translation [78,79,80] but, in human cells, La has been shown to bind and repress 5'TOP translation [81]. There is evidence that La switches between binding tRNAs and 5'TOPs in response to its phosphorylation status. Specifically, on phosphorylation of La by the protein kinase Casein Kinase 2 (CK2) at serine 366, La binds and activates tRNA assembly but when dephosphorylated it represses the translation of TOPs such as the 60S ribosomal protein subunit L37 (rpL37) [82].

Little is known about the regulation of La expression. Microtubule-based transport of La within sensory axons has been shown to be dictated by its SUMOylation status [83]. Phosphorylation of La is key to its activity and perhaps also its ability to recognise substrates [20] and known upstream kinases so far include CK2 and AKT1 [84,85].

## 4. Genuine La and Cancer

The oncogenic role of La has so far been attributed to its IRES-mediated target genes, particularly BiP, MDM2 and Cyclin D1 which are themselves independently associated with malignancy [86,87,88]. Sommer *et al.* [54] demonstrated that aberrant over-expression of La drives cell migration and invasion and that elevated La levels are observed in squamous cancers of the head and neck and cervix. Interestingly, La seems to have a tissue-specific mRNA interactome, upregulating MDM2 in head and neck cancer but Cyclin D1 in cancer of the cervix [41,54]. In hepatocellular cancer cells, La has been shown to drive epithelial to mesenchymal transition (EMT) through expression of the IRES-mediated target Laminin B1 [36]. In patients with myeloproliferative diseases that carry a JAK2 mutation (V617F), La-regulated translation of MDM2 drives cell proliferation, over-riding the p53 response to DNA damage by degrading p53. The observed functional inactivation of p53 makes these cells uniquely sensitive to MDM2 inhibitors such as Nutlins [55].

## 5. LARP1

There are two paralogues of LARP1 genes in humans: LARP1 or 1a (positioned at chromosome 5q34) and LARP1b or LARP2 (at 4q28) encoding 1096 and 914 amino acid proteins, respectively. Although there is some similarity between LARP1a and b (60% homology and 73% positivity), LARP1a is the more abundantly expressed and gives the strongest knockdown phenotype [44]. There has been no published work on LARP1b and publications describing LARP1, including this one, refer exclusively to LARP1a. Like La, LARP1 is present both in the nucleus and cytoplasm but, unlike La, LARP1 is predominantly cytoplasmic. In addition, LARP1 carries a highly conserved “DM15 region” (or “LARP1 motif”; here, we will refer to it as the DM15 region to avoid confusion with the LAM and La module). LARP1 was first identified in *Drosophila*, and shown to be required for embryogenesis [26] oogenesis, spermatogenesis, formation of the mitotic spindle poles, successful segregation of mitochondria [89] and cell cycle progression [46]. Although LARP1 has been identified as a host factor for influenza A and HIV infection, it has no described function in transcription but is highly influential in mRNA translation [90,91]. LARP1 was the first of the LARP family to be shown to bind PABP in an RNA-independent manner [46]. PABP is implicated in mRNA translation [92], and knockdown of LARP1 was associated with a 15% fall in overall protein synthesis, and an increase in hypophosphorylated 4E-BP1 [44] implying involvement in cap-mediated translation. This was supported by evidence that LARP1 was complexed with 5' cap-binding components [37,44,45].

**Figure 2:**
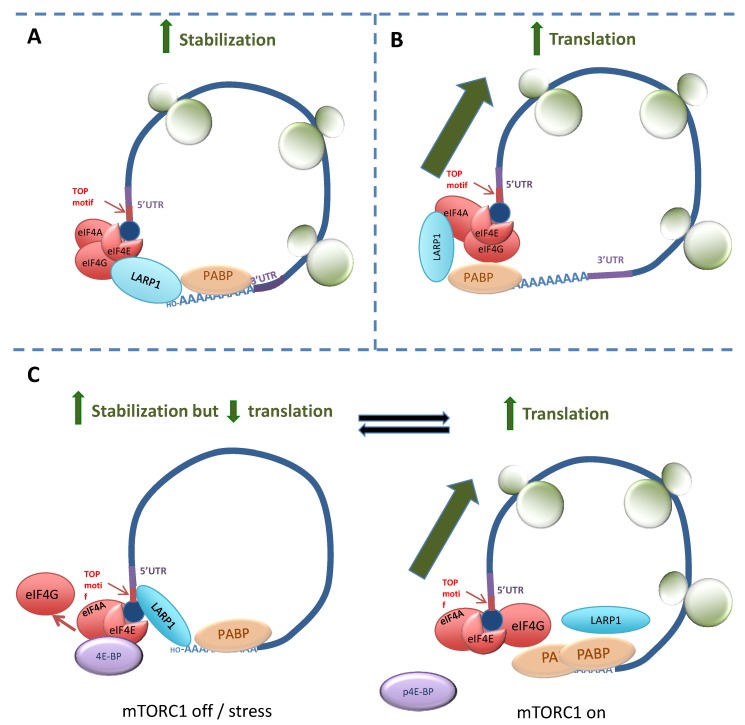
Proposed mechanisms for the interaction between LARP1 and 5'TOPs. (**A**) Aoki *et al.* (2013) [45] demonstrated that LARP1 independently binds the terminal adenosine of the poly(A) tail of mRNA and, by simultaneously interacting with 5'cap components, circularises mRNA of 5'TOP-bearing transcripts to enhance their stability; (**B**) Tcherkezian *et al.* (2014) [37] showed LARP1 as associating with 5' cap components via its interaction with PABP. LARP1 is complexed with Raptor, which enhances 5'TOP translation, but it is unclear whether this is functionally distinct from its association with PABP/eIF4F; (**C**) LARP1 in the mTORC1-regulated phosphorylation-dependent switch mechanism described by Fonseca *et al.* (2015) [38]. Upon mTORC1 inhibition or cell stress (“mTORC1 off”), LARP1 displaces eIF4G and binds both to the 5'TOP sequence and the poly (**A**) tail of TOP mRNA to create a stable “closed loop” which is translationally inactive. When mTORC1 is active (“mTORC1 on”) 4E-BP1 is phosphorylated whereupon eIF4E is released and returns to the eIF4F complex. At the same time, LARP1 (complexed with Raptor) is activated by mTORC1 and binds PABP. The mRNA then assumes an “open-loop” or translationally active conformation.

More recently, LARP1 has been identified as the putative “missing link” between mTORC1 and ribosome biogenesis [93]. Mammalian target of rapamycin (mTOR) is a kinase that is a central regulator of protein synthesis in response to changes in nutrients, energy supply or reactive oxygen species [94]. The mTORC1 complex, comprised at least of mTOR, Raptor, MLST8, PRAS40 and DEPTOR, has long been known to control the translation of 5'TOPs [95]. Although translation of TOP mRNAs is highly sensitive to the mTORC1 inhibitor rapamycin, a direct interaction between mTORC1 and the TOP mRNAs had not been shown [95]. However, recent studies have identified LARP1 as linking mTORC1 signalling to 5'TOP activity. A summary of three proposed mechanisms is shown in Figure 2. The first, published in 2013 by Tohru Natsume’s group [45], identified LARP1 as directly interacting with the poly(A)-tail of 5'TOP mRNAs (Figure 2A). Validating their findings using LARP1-RNA immunoprecipitates from HEK293 cells, they demonstrated an enrichment of poly(A) bearing transcripts and absence of mRNA species lacking poly(A) tails (such as histone mRNA and rRNA). They hypothesised that, by simultaneously binding the 5'cap and the poly(A) tail of actively translating 5'TOP mRNA transcripts, LARP1 stabilises their circular conformation to sustain protein synthesis.

In 2014, Philippe Roux’s group identified that the interaction between LARP1 and PABP was mediated by its DM15 region. They postulated that the interaction between LARP1 and 5' cap (eIF4F) proteins was an indirect effect of its interaction with PABP, a known associate of eIF4F components [37,96]. Using sucrose density gradient centrifugation to fractionate polysomes from HEK293 cells, LARP1 was shown to be associated with pre-polysomal (monosome and ribosome) and polysomal mRNA fractions but shifted to the pre-polysomal fractions on treatment with a dual mTOR/PI3K inhibitor. The effect of LARP1 depletion was assessed on a selection of TOP mRNAs where it was shown to prevent their translation. Consistent with this, proteins encoded by TOP mRNAs were less abundant in protein lysates from LARP1-depleted cells. These results suggested LARP1 had a positive effect on protein synthesis overall, as was previously reported by Aoki and Burrows *et al.* [44,45]. As the authors also demonstrated an association between LARP1 and Raptor, they hypothesised that LARP1 was an mTOR-dependent activator of 5'TOP translation and cell proliferation (Figure 2B).

Bruno Fonseca and co-workers came to an opposing view in their research published in 2015 (Figure 2C) [38]. Like the Roux group, they demonstrated interactions between LARP1 and PABP and Raptor in HEK293 cells but concluded that LARP1 *negatively* regulates TOP translation. Whilst they showed an interaction between LARP1 and Raptor, treatment using the mTORC1/2 inhibitor Torin or cell stress by nutrient deprivation caused LARP1 to displace eIF4G from the eIF4F complex, become complexed to the polypyrimidine sequence of 5'TOPs and to repress their translation. They found LARP1 was associated with Raptor in conditions of normal mTORC1 activity and hypothesised that, upon phosphorylation of 4E-BP1 by mTOR, eIF4E returns to the eIF4F complex and (possibly as a result of phosphorylation by mTOR) LARP1 binds PABP. This allows 5'TOP mRNA to assume an “open” conformation stimulating translation. As their hypothesis that LARP1 is an overall inhibitor of protein synthesis contradicts findings made by others (Burrows *et al.* [44], Aoki *et al.* [45] and Tcherkezian *et al.* [37]), the significance of Fonseca *et al.*’s findings remains uncertain. A possible explanation for these discrepancies is that, like La, LARP1 switches function in response to its phosphorylation status and can thus either stimulate or inhibit translation. Alternatively, LARP1 may have a dual function by acting as a regulator and scaffold. As a regulator, LARP1 may prevent eIF4F assembly on TOP mRNAs by directly binding the TOP oligopyrimidine motif via its DM15 region, but at the same time acting as a scaffold that brings mTORC1 to these proteins for their activation (*i.e.*, 4E-BP1 phosphorylation, LARP1 phosphorylation and removal). As yet, it is unclear whether this dual function is preserved in non-TOP mRNAs.

## 6. LARP1 and Cancer

By referencing *in-silico* databases like Oncomine [97], it is apparent that LARP1 is over-expressed in the majority of epithelial malignancies compared to their adjacent normal tissues. In several cancers, the relationship between LARP1 expression and clinical outcome has been studied. Levels of LARP1 protein correlate with increasing disease progression in cervical cancer where there are stepwise elevations in LARP1 expression through the pre-invasive stages (CIN1-3) and into invasive disease [44]. In hepatocellular cancer (HCC), high levels of LARP1 protein in tumour tissue correlate with approximately 35% increased risk of death by five years (compared to low levels) [56] and with tumour size, survival time and Child-Pugh score. However, both HCC and squamous cancer of the cervix are associated with chronic viral infection: hepatitis B/C and herpes simplex, respectively. With some evidence that LARP1 is a viral host factor, upregulation of LARP1 in these tumours could perhaps reflect ongoing viral infection rather than an active role in the cancer process. However, in the HCC patients there was no correlation between Hepatitis B surface Ag status (a marker of infection) and LARP1 expression, although their hepatitis C status was unknown [56]. Additionally, levels of LARP1 have also been noted to be high in non-virally associated cancers such as prostate and breast cancer. In prostate cancer cells, tumour migration is attenuated upon LARP1 knockdown and LARP1 expression is negatively regulated by microRNAs mi-26a or b [57]. In breast cancer, RNA-sequencing and high throughput software analyses of tissue samples revealed the presence of a novel LARP1 splice variation in 4/6 non-triple negative cases. This variant was also detected in MCF7 cells although its prognostic relevance is unknown [98].

In HeLa (cervical cancer) cell lines, loss of LARP1 by transient RNAi, stable transfection or lentiviral knockdown causes a proportion of them to undergo apoptosis [43]. This is in contradiction to the findings in non-malignant immortalised (such as HEK293) cell lines but also in two malignant cell lines HEC-1B and A549 in which G_0_/G_1_ arrest and attenuation of proliferation were the predominant phenotypic changes observed after LARP1 depletion [37]. It is noteworthy that HeLa and PC9 cell lines are TP53 null or mutated respectively whilst HEK293, HEK-1B and A549 lines are TP53 wild type, implying the response to LARP1 knockdown may be p53-dependent. This is worthy of further investigation. An RNA-immunoprecipitation and cDNA microarray (RIP-chip) conducted in HeLa cells revealed LARP1 is in complex with approximately 3000 mRNAs [43]. It has a similarly large interactome in HEK293 cells (unpublished data) but, in the cancer interactome, although TOP mRNAs are present, there is a preponderance of cancer-sustaining transcripts including mTOR and other components of its signalling pathway. In cancer cells, upregulation of LARP1 increases cell migration, invasion, EMT and tumourigenesis [44]. This implies that LARP1 has a distinct interactome in cancer compared to non-malignant cells that could result from conformational changes to LARP1 driven by upstream signalling events, partner protein interactions or associations with other RBPs, micro or non-coding RNAs. Dr Berman’s group has identified that the DM15 region of LARP1 can dimerise to generate a hypothetical pocket capable of binding single stranded RNA [52]. This dimerisation may be concentration-dependent, and thus more prevalent in cancer cells with higher levels of LARP1. This has yet to be addressed experimentally but, as a hypothesis, could explain the different interactome observed in cancer cells.

## 7. LARP4a

There have been two paralogues of LARP4 identified to date: LARP4a and LARP4b (previously called LARP5) at genomic locations 12q13.12 and 10p15.3, respectively [19]. LARP4a and 4b share 38% amino acid identity with each other overall but 74% identity between their La modules. They are the most divergent of the LARPs from La protein. LARP4a has multiple predicted splice variants of unknown functional significance [45]. It stimulates mRNA translation, at least in part through its interactions with the scaffold protein Ribosome-Associated Receptor for Activated C Kinase 1 (RACK1), with cytosolic PABP, and with mRNAs via their polyadenylate tails [23]. The full LARP4a mRNA “interactome” has yet to be characterised but, unlike the other LARPs, LARP4a does not appear to have an additional C-terminal motif that might extend its binding repertoire. It does, however, have a non-canonical PAM2 domain (the PAM2w) within its N-terminus that is believed to act in concert with the La domain to recognise and bind PABP [23,99].

## 8. LARP4a and Cancer

Of all the LARPs, LARP4a has the most tenuous link to cancer. Unlike La, LARP1 and LARP6, knockdown of LARP4a in cancer cells promotes rather than inhibits cancer cell migration [58]. This has been observed in one prostate cancer (PC3) cell line so far [58] and is possibly context-specific, so further characterisation is required. Perhaps, loss of LARP4a induces RACK1 to interact with pro-migratory partners. However, placed in the context of the other LARPs, it seems unlikely that LARP4a has such a limited binding repertoire and transcriptome-wide analysis is needed.

## 9. LARP4b

Angenstein *et al.* [28] first reported LARP4b as being associated with PABP and capable of binding poly(A)-mRNAs in rat neurogenic cells. Schäffler and colleagues [39] demonstrated that LARP4b stimulates translation and co-sediments with 80S ribosomes, particularly the 40S ribosome component and this is mediated either directly or via its interaction with RACK1. During translation, mRNA is circularised to allow efficient translation re-initiation after the ribosome reaches the 3' end of mRNA. The authors speculated that LARP4b stabilises mRNA in a circular conformation by binding 3' associated PABP and 5' associated RACK1 to drive protein synthesis. LARP4b has yet to be shown to directly interact with mRNA, but extrapolating from its paralogue LARP4a, this seems likely. Although an interactome for LARP4b in either non-malignant or malignant cell lines has yet to be described, Zhang *et al.* [59] identified an elevation in mRNA levels of the tumour suppressor cell cycle factors p16 and p19 and the transcription factor C/EBPα on LARP4b knockdown, suggesting a role in suppressing their expression and supporting a pro-migratory and cell cycle stimulatory role for LARP4b.

## 10. LARP4b and Cancer

There has been no published association between LARP4b and cancer. However, in work published by Zhang *et al.* [59], lentiviral suppression of LARP4b in a myeloid leukaemia (MLL-AF9) model reduced leukemic stem cells, attenuated their self-renewal capacity and caused them to undergo a cell cycle arrest.

## 11. LARP6

LARP6 was first identified in moth species where it was associated with the programmed cell death of intersegmental muscles that occurs during the developmental transition from pupa to adult. The gene was originally named Acheron (Achn) after the river of death in Greek mythology [29,100]. In addition to its N-terminal La module, LARP6 carries a conserved SUZ-C motif in its extreme C-terminus that is present in other RBPs and believed to contribute towards mRNA substrate recognition [101]. LARP6 binds two regions of the 5' stem-loop of alpha 1 (I), alpha 2 (II) and possibly also alpha 1 (III) collagen mRNAs to regulate their localised translation. It also binds vimentin intermediate filaments, RNA helicase, STRAP and non-muscle myosin, factors believed to sustain collagen alpha 2 production alongside, so that heterotrimeric collagen I fibres are generated during reparative or reactive fibrosis [33,34]. The ability of LARP6 to recognise and bind collagen mRNA was previously attributed to its “bipartite” RNA binding domain comprising its La motif and a 40 amino acid sequence N-terminal to it that is absent from other LARPs [19,40]. More recently, its substrate binding has been attributed to the structure and composition of the inter-linker domain lying between the La motif and the RRM within its La module [102].

LARP6 is predominantly expressed in neurons, striated skeletal and cardiac muscle. It is required to determine the fate of nascent muscle fibres (myoblasts), either to undergo differentiation, proliferation or apoptosis in response to ECM signals [29]. LARP6 also influences cell adhesion, morphology and structure as well as cytoskeletal organisation. This is either through the translation of, as yet unknown, target mRNAs or as an indirect result of collagen I upregulation in myofibroblasts causing a corresponding increase in non-muscle myosin synthesis [34]. Using human aortic smooth muscle cells, Blackstock and co-workers identified LARP6 as being activated downstream of insulin-like growth factor 1 (IGF-1) via PI3K/AKT pathway signalling [33]. LARP6 shuttles between the nucleus and cytoplasm and participates in the nuclear export of collagen mRNAs [40]. Weng *et al.* [47] demonstrated that LARP6 binds to the developmental transcription factor CASK-C, a novel CASK/Lin-2 isoform, and forms a complex with Id (inhibitor of differentiation) transcription factors [47]. It has also been shown to act upstream of the transcription factor MyoD which regulates muscle development [29]. Weigand *et al.* [103] demonstrated there were two isoforms of LARP6 and showed that, in endothelial cells exposed to hypoxia, the shorter isoform 1 was down-regulated, whereas the full-length isoform 2 was not. Although it has also been linked to integrin expression, the principal role of LARP6 in non-malignant cells appears to be in collagen I synthesis. As type I collagen is the most abundant protein in the human body (making up 25%–30% of its total protein content), it is possible that LARP6 has evolved purely to execute this role, or perhaps collagen transcripts are components of a wider RNA-operon involved in muscle development and regeneration. However, a full analysis of the LARP6 interactome has yet to be performed.

Stefanovic and Stefanovic [40] developed a fluorescence polarisation high throughput screen to identify inhibitors of the interaction between LARP6 and the 5' stem loop of collagen, with the aim of developing them as antifibrotic agents. Using this assay, a number of candidate drugs have been identified but these have yet to reach the clinic.

## 12. LARP6 and Cancer

LARP6 is highly expressed in the myoepithelial cells of the mammary gland and is upregulated in basal-like invasive ductal carcinomas of the breast. Ectopic expression of LARP6 in MDA-MB-231 breast cancer cells enhances their proliferation, lamellipodia formation and invasion as well as their upregulated expression of MMP-9 and VEGF [60]. *In vivo*, using a human tumour xenograft model, LARP6 acts as an oncogene, enhancing angiogenesis and tumour growth. These features are dependent on the ability of LARP6 to enter the nucleus and are attenuated when its nuclear localisation signal is removed [60]. The reason for its functional dependence on nuclear shuttling is unclear, as the protein is predominantly cytoplasmic. It is possibly related to role in the transcription of MMP-9 and VEGF, as LARP6 has been shown to drive MMP-9 activity from its promotor [60]. This is reminiscent of the observation that over-expression of LARP6 caused upregulation of the transcription factor MyoD in zebrafish embryos and mouse myoblasts [47].

## 13. LARP7

The domain structure of LARP7 is very closely related to that of Genuine La. Like La, LARP7 binds RNA polymerase III transcripts but replaces La protein in specifically recognizing the UUU-3'-OH of a single RNA pol III transcript, the 7SK snRNA in animals, or telomerase RNA in protista [35]. Human 7SK RNA is an abundant non-coding RNA that regulates mRNA metabolism by controlling the activity of the positive transcription elongation factor b (P-TEFb), a cyclin dependent kinase required for RNA polymerase II transcription elongation [104]. LARP7 binds to uridines at the 3'-terminus of 7SK RNA and together with the 5' capping protein MePCE, circularises it and creates a stable 7SKsnRNP structure that is protected from exonuclease digestion. As with La, the recognition for the 3' terminus by LARP7 is sequence specific [48]. The complex suppresses the P-TEFb complex, consisting of CDK9 and Cyclin T1. Thus, LARP7 indirectly suppresses mRNA transcription [30]. LARP7 recognises and binds a 3'-hairpin loop near the U-rich tail of human 7SK snRNA as well as the C320-U321 internal bulge [48]. These interactions are dependent on the La module in tandem with the RRM2 [105]. By regulating P-TEFb activity and thus the rate of PolII elongation and recruitment of splicing factors to its C-terminal domain, LARP7 also indirectly influences alternative splicing [106].

## 14. LARP7 and Cancer

Loss of LARP7 activates transcriptional elongation. This is because without LARP7, 7SK RNA is degraded and P-TEFb protein is released from the catalytically inactive 7SK snRNP complex and is shifted towards an active state [61]. P-TEFb is recruited to chromatin by the bromodomain protein Brd4 where it promotes transcription and cell cycle progression [107]. Other cellular proteins also recruit P-TEFb to active transcription sites, such as the oestrogen and androgen receptors and MyoD [107]. Microsatellite-instability induced frame-shift mutations resulting in truncations to the RRM2 region of LARP7 (required for its interaction with 7SK RNA) have been detected in a significant proportion of gastric cancer cases [108]. A study by Cheng *et al.* [63] suggest that LARP7 is a potential tumour suppressor in gastric cancer and that LARP7 down-regulation occurs early during gastric tumorigenesis and may promote it via P-TEFb dysregulation.

Knockdown of LARP7 in mammary epithelial cell line MCF10A disrupts cell polarity and morphological differentiation [61]. More recently, short hairpin silencing of LARP7 in MCF10A cells has been shown to upregulate the P-TEFb mediated expression of EMT and metastasis genes (such as Slug, ZEB2 and Twist1) resulting in enhanced tumour progression and metastasis [62]. Expression of LARP7 is downregulated in invasive human breast cancer and higher levels of the protein are associated with improved overall and longer recurrence-free survival. Interestingly, expression of LARP7 protein is lost from tumour tissue during cancer progression [62].

## 15. Conclusions

The “archetypal LARP” Genuine La was the first to be discovered. It was shown to be a canonical component of transcription via its effects on tRNA maturation and polymerase III transcript stability. As the other LARPs have subsequently been described, it has become apparent that La is not in fact archetypal and that the family, with the exception of La and LARP7, is more predominantly involved in mRNA metabolism. With combined insights from structural and evolutionary biologists, this functional diversity has been attributed to subtle amino acid variations within each La module, as well as the inclusion of PABP-binding domains (in the case of LARP4a/4b) or other C-terminally placed motifs, such as the DM15 region of LARP1. When present, these modifications appear to broaden the binding repertoire of LARPs to include mature messenger RNAs and PABP and thus function in mRNA metabolism. There remain many unanswered questions about the activity of the LARPs, a few of which we will address here.

Despite identifying a number of mRNAs bound to LARPs, there are no defined LARP-recognition or “USER” (untranslated sequence element for regulation) sequences within these target mRNAs. The LARPs seem capable of exerting binary control over the half-lives of their targets, stabilising some but destabilising others, dictated perhaps by their USER sequence or conformation. Once bound to LARPs, the exact fate of these LARP-bound transcripts is also unknown. LARP4b and LARP1 have individually been located in cytoplasmic stress granules and processing bodies (P bodies), suggesting that mRNAs are tracked there for protection or enzymatic digestion, respectively [39,109,110]. It is possible that LARPs regulate this tracking once bound to their targets. It is also likely that LARPs cooperate with other RBPs, proteins, ncRNAs or miRNAs. In the plant species *Arabidopsis*, LARP1 complexes with XRN4 to target mRNAs for degradation during heat stress [109] and cooperation with miRNAs has already been described for other RBPs such as HuR and miR-122 [111]. As there is considerable overlap in phenotype between them it is possible that two or more members of the LARP family work together. These questions can be addressed by conducting RNA-capture and sequencing techniques such as Photoactivatable-Ribonucleoside-Enhanced Crosslinking and Immunoprecipitation (PAR-CLIP), individual-nucleotide resolution Cross-Linking and ImmunoPrecipitation (iCLIP) and most recently Degradation-Optimised RNA-Immunoprecipitation and Sequencing (DO-RIP-Seq) for each and every LARP. This would clarify the USER sequences or structures within their mRNA targets and define their mRNA interactomes. Equally, the development of LARP-knockout mice will enable further characterisation of the impact of each of the LARPs towards normal and cancer cell physiology.

So far, the PI3K/AKT/mTOR pathway has been implicated in the phosphorylation of La, LARP1 and LARP6, but other upstream drivers for these and the other LARPs remain unknown. LARP1 appears to be the family member most intimately involved with mTOR signalling, being bound to Raptor and controlling the stability of many mRNAs within the mTOR pathway, including mTOR itself. As components of the mTORC1 pathway are dysregulated in approximately 80% of malignancies [112], it is perhaps inevitable that the LARPs are also implicated in cancer. La, LARP1 and LARP6 are upregulated and associated with tumour invasion and migration whereas LARP7 and possibly LARP4a are down-regulated in malignancy and associated with progression and metastasis. For LARP1 (and maybe other LARPs), the target mRNAs differ between non-malignant and malignant cell lines. In the malignant cells in which it has been characterised, the LARP1 interactome includes multiple cancer-sustaining targets especially those involved in cell survival. Although no clinically significant LARP1 mutations have yet been identified in cancers, a LARP1 splice variant has been observed in breast cancer. It is possible that splicing alters the alignment of RNA binding modules or linker regions within LARPs changing their conformation and their selected substrates. As has been observed with La, phosphorylation or other post-translational modifications of the LARPs might enable them to engage with certain protein-partners, or even homo or hetero-dimerise to generate new substrate-binding surfaces. The recent identification of homo-dimerisation at the DM15 region of LARP1 supports this hypothesis.

Although La, LARP1 and LARP6 are regulated by mTORC1, they have functional properties that make them distinct from mTORC1 in the scope of their activity and influence. Unlike mTORC1, LARP1 contributes directly to 5'TOP signalling, but also controls the stability of cell survival genes and components of the other signalling pathways [37,38,43]. This has important clinical implications. MTORC1 inhibitors are already widely used anti-cancer agents but have demonstrated only modest efficacy in all but a few rare cancers [113]. This has been attributed to various factors including their inability to directly regulate 5'TOPs and the activation of feedback pathways acting via PI3K and AKT that promote cell survival [113]. As mTORC1 has a central role in normal cellular homeostasis, inhibitors cause multiple side effects such as myelosuppression, hypertension, hyperglycaemia and pneumonitis causing a high treatment drop-out rate amongst cancer patients [114]. LARP1 is potentially a more desirable therapeutic target. It is expressed at high levels in cancer cells and has a binding repertoire that includes mRNAs encoding cancer pathway proteins and oncogenes. However, a broader understanding of LARP1 biology is required before embarking on the development of LARP1 inhibitors. LARP6, as well as post-transcriptionally driving collagen synthesis which is an essential cytoskeletal component of the migrating cancer cell, also regulates gene transcription and LARP4b regulates the transcription factor C/EBPα. That LARP6 and 4b primarily affect translation but also regulate transcription suggests they are involved in feedback coupling between the two processes. LARP6 inhibitors are currently being developed for the treatment of fibrotic disease, but because of their direct influence on gene transcription, may also have utility in cancer treatment.

Therapies to attenuate pathological upregulation of La, LARP1 or LARP6 are likely to have profound anti-proliferative and pro-apoptotic effects in cancer cells. Agents that stabilise LARP7 may also have clinical utility. Currently, CDK9 inhibitors are being explored as anti-cancer therapies because the kinase stabilises the P-TEFb complex and drives PolII transcription elongation of various oncogenic mRNAs. However, CDK9 inhibitors like flavopiridol cause off-target effects due to inhibition of other CDKs [115]. A LARP7-centred approach may have a more specific effect and this is worthy of further investigation.

We have shown here that the LARPs are a fascinating family of proteins that each make significant, but significantly different, contributions to gene expression. However, the picture is incomplete and there are many gaps in our understanding of LARP biology. Collectively, it is becoming apparent that most LARPs contribute to cancer behaviour and may themselves be novel therapeutic targets to explore in the future.
